# Team science training for clinical and translational Scientists: An assessment of effectiveness

**DOI:** 10.1017/cts.2025.10088

**Published:** 2025-06-30

**Authors:** Anne Mook, Verena Knerich, Goldie Komaie, Lisa Cicutto, Jennifer Cross

**Affiliations:** 1 Colorado Clinical and Translational Sciences Institute, Workforce Development, Fort Collins, CO, USA; 2 Institute for Research in the Social Sciences, Colorado State University, Fort Collins, CO, USA; 3 Department of Sociology, Colorado State University, Fort Collins, CO, USA; 4 PBL Environmental Assessment Agency, The Hague, Netherlands; 5 Ludwig Maximilian University, Institute of Intercultural Communication, Munich, Germany; 6 The Evaluation Center, School of Education & Human Development, University of Colorado Denver, Denver, CO, USA; 7 Clinical Science and College of Nursing, University of Colorado | Anschutz Medical Campus, Aurora, CO, USA; 8 Community Outreach and Research, National Jewish Health, Denver, CO, USA

**Keywords:** Evaluation, clinical and translational science, training, collaboration, learning outcomes

## Abstract

**Introduction:**

Effective interdisciplinary collaboration is essential for addressing complex clinical and translational research challenges. This paper presents and evaluates a structured team science training program developed by the Colorado Clinical and Translational Sciences Institute (CCTSI), while also introducing and validating a novel assessment tool used to measure changes in key teaming competencies.

**Methods:**

We evaluated the effectiveness of this program between 2020 and 2022 using pre- and post-program surveys (*N* = 221). Our evaluation tool was designed to capture familiarity with teaming concepts and the frequency of applying collaborative practices. Principal component analysis (PCA) was applied to validate the grouping of these competencies, and paired *t*-tests were used to measure changes over time.

**Results:**

PCA revealed three distinct components of team science competencies: Team Planning, Managing a Team, and Interpersonal Relations, all demonstrating strong internal reliability. Participants showed statistically significant improvements (*p* < 0.05) in all three domains. Gains were robust in Team Planning and Managing a Team, emphasizing structured tools and practices. Although improvement was also observed in Interpersonal Relations, the overall gains were smaller.

**Conclusions:**

These findings highlight the self-reported value of Team Science Training programs in CTR settings. The TEAMS instrument described in this manuscript offers a novel approach for CTSAs to evaluate their Team Science training programs. Future applications could include longitudinal tracking and integration metrics to support future program planning, particularly fostering interdisciplinary collaboration and team integration.

## Introduction

As scientific challenges become more complex, solutions increasingly require interdisciplinary approaches, necessitating team science as a critical strategy for advancing discovery and translating research into practice. The need for effective collaboration is particularly pronounced in clinical and translational research (CTR), where bridging the gap between basic science and real-world healthcare applications requires high-performing research teams. An extensive body of literature documents that ineffective collaboration causes poor performance, dissatisfaction, and high turnover of team members [1,2]. Despite the importance of effective teaming in science, training in team science competencies is only slowly being integrated into post-baccalaureate educational programs [3–6]. The Science of Team Science field has expanded over the past two decades to study and improve scientific collaboration [7,8]. The field equally strives to research and document factors associated with success in science teams, as well as build a body of literature on impactful training and team interventions to improve team effectiveness and the capacity of integrative teams to solve complex problems [8].

Scientists increasingly recognize the requirements, challenges, and opportunities to enhance collaborative capacity for interdisciplinary projects [6,9]. Core competencies of collaborative capacity include (1) the planning necessary to build and grow a team, (2) the management required to monitor progress and stay on track, and (3) the fostering of effective Interpersonal Relations among team members needed for mutual learning and social support [5,12]. Many Clinical and Translational Science Award (CTSA) Hubs and interdisciplinary research centers have introduced Team Science training programs to strengthen these competencies, particularly in biomedical and health research [4,13,14]. Yet, despite the proliferation of training programs, there remains little empirical evidence demonstrating their impact on actual teaming behaviors, scientific outputs, or research impact [4,5,15].This paper introduces and validates an assessment tool for Team Science competencies and reports findings from its application in evaluating a structured training program. Unlike research on traditional educational domains, such as reading and mathematics, where learning mechanisms are well studied, the effects of Team Science training on real-world research teams remain poorly understood. For example, regarding reading ability in elementary school, we know that children with higher baseline reading skills tend to improve their reading skills more quickly, a phenomenon known as the Matthew effect. The explanation is that children who excel at reading receive praise for their abilities, enjoy reading, and consequently read more and learn faster [16,17]. On the other hand, there is also the Dunning–Kruger effect, which is well-documented among students in medicine and psychology, where novices tend to overestimate their competence because they lack the skills to accurately recognize deficient performance [18,19]. For Team Science, it remains unclear whether a similar Matthew Effect exists, where individuals with stronger teaming skills thrive and continue to develop, or whether a lack of awareness about ineffective teaming practices prevents researchers from recognizing their deficits until they are exposed to structured training. On the one hand, good team players may enjoy the collaborative process, be successful in science teams, learn from their experiences, and feel encouraged to apply their collaborative skills to more team-based research. On the other hand, researchers may be unaware of their lack of teaming competencies. They may not recognize this deficiency or its impact until they are exposed to team science frameworks and experience better teaming practices.

This study evaluated the effectiveness of the Colorado Clinical and Translational Science Institute’s (CCTSI) Team Science training program using pre- and post-program surveys. The primary objectives were twofold: (1) to validate the grouping of Team Science competencies – namely, Team Planning, Managing a Team, and Interpersonal Relations – within the newly developed Teaming Evaluation and Assessment for Multidisciplinary Science (TEAMS) instrument, and (2) to assess, through self-reports, the program’s impact on enhancing teaming capacity in clinical and translational science teams. The TEAMS instrument was designed to measure key competencies in team science. By analyzing pre- and post-program survey data, the study aimed to determine whether the training effectively improved participants’ self-reported competencies in these three domains. The findings offer valuable insights into participants’ experiences with the Team Science training program in Colorado and highlight areas for further research to enhance the effectiveness of such training initiatives.

## Methods

### Team science program

The CCTSI’s Team Science training program was provided three times a year. The program began with a self-paced online Teaming 101 web module, comprising six modules featuring 20-minute lecture videos, scenario videos, and a short assessment to evaluate participants’ understanding of core concepts. Completion of the Teaming 101 web module was mandatory before attending any of the six virtual (Zoom) interactive workshops. This prerequisite ensured that all participants had a foundational understanding of team science concepts before engaging in interactive discussions. After completing the Teaming 101 web module, participants could register for any workshop in any order. The six workshops covered key domains of Team Science, focusing on building relationships and trust (Workshop 1), setting expectations (Workshop 2), shared language and vision (Workshop 3), collaborative knowledge creation (Workshop 4), change management and conflict resolution (Workshop 5), and leadership (Workshop 6). Each workshop addressed specific competencies, such as psychological safety, authorship agreements, reflexivity, power dynamics, and conflict management (see Table 1 for a detailed breakdown of workshop topics).

**Table 1.** Description of the topics covered in the six interactive virtual workshops facilitated on zoom

Workshop	Key Topics Covered	Literature
**Workshop 1: Building Relationships and a Team**	• Types of trust • Psychological safety • Social sensitivity • Inclusivity and diversity • Building rapport and having fun	[28] Putman & Paulus (2009) [29] Edmondson (1999) [30] Dietsch et al. (2021) [31] Bender et al. (2012) [32] Brown et al. (2021)
**Workshop 2: Setting Expectations**	• Problems of unacknowledged differences • Turn-taking • Nonverbal overload • Communication plans • Authorship agreements	[33] Woolley et al. (2010) [34] Rasmussen et al. (2023) [35] Mohammed & Harrison (2013)
**Workshop 3: Building a Shared Language and Vision**	• Unacknowledged differences • Cross-disciplinary research and translational science • Reflexivity and perspective-taking • Community agreements • Shared visioning	[36] Nissani (1995) [37] Eigenbrode et al., (2007) [38] Salazar et al., (2029)
**Workshop 4: Collaborative Knowledge Creation**	• Creative thinking process • Team innovation • Creativity techniques • Collaborating with community partners	[39] McFadzean, (2000) [40] Hubbs et al. (2020) [41] Rinkus et al. (2021)
**Workshop 5: Change Management and Conflict Resolution**	• Conditions for change • Power dynamics • Strategies for saying no • Conflict management	[42] McKeown (2014) [43] Thomas & Kilmann (2007) [44] Souza (2018)
**Workshop 6: Leadership for All Team Members**	• Leadership theories • Leaders and followers • Giving and receiving feedback • Types of mentorships	[45] Thompson & Vecchio (2009) [46] Ford & Harding (2018) [47] Mullen & Klimaitis (2021)

All workshops employed evidence-based active learning strategies, including small-group discussions, polls, role-playing, skill practice, problem-solving, group ideation, and reflection [20]. After each workshop, participants received a written summary of key content and takeaways for future reference.

### Target audience

The program was developed for and open to all early-career investigators and clinical research professionals. Specifically, early-career investigators were defined as pre- and post-doctoral students, instructors, and assistant professors. The CCTSI community includes three University of Colorado (CU) campuses (CU Boulder, CU Denver, CU Anschutz Medical Campus), Colorado State University, and healthcare partners (Children’s Hospital of Colorado, Denver Health, National Jewish Health, University of Colorado Health, and the Rocky Mountain Regional VA Medical Center). Potential participants were informed of the program through CCTSI newsletters, email blasts, and the CCTSI website.

### Pre- and post-program assessments

The University of Colorado Institutional Review Board deemed the study exempt and advised that these activities are program evaluation rather than human subject research. As part of the registration process, participants were required to complete a registration form, which collected their demographic characteristics. Participants then received the URL link through Qualtrics to complete the Teaming 101 web module. A pre-post survey assessed and measured changes in familiarity and frequency of use in applying teaming concepts. The pre-program survey was administered as part of the Teaming 101 web module. Post-program surveys were sent to participants one month after attending all six workshops.

To systematically assess the effectiveness of the CCTSI Team Science training program, we developed the Team Evaluation and Assessment for Multidisciplinary Science (TEAMS) instrument. This tool evaluated key competencies for effective team science, structured around three core domains: Team Planning, Managing a Team, and Interpersonal Skills. The development process involved identifying specific behaviors and strategies pertinent to each domain, ensuring that the instrument accurately captures the multifaceted nature of team-based scientific collaboration. By aligning the TEAMS instrument with the objectives of the training program, we aimed to provide a reliable measure of participants’ competencies and the program’s impact on enhancing team science skills. In the pre- and post-program TEAMS surveys, participants were asked to self-report their familiarity with Team Science competencies for Team Planning, including building a shared language, establishing a shared vision, setting ground rules, and creating team charters. Additionally, participants were asked to report the frequency of using team science strategies when working on research projects for two other components: *Managing a Team*, which involves creating meeting agendas, monitoring progress, establishing authorship agreements, and incorporating others’ perspectives, and *Interpersonal Skills*, which includes perspective-taking, facilitating awareness and exchange, acknowledging and including diversity, and promoting equal turn-taking. Familiarity with the Team Planning and Managing a Team concepts was assessed using a five-point Likert scale, with responses ranging from 1 (“Strongly Disagree”) to 5 (“Strongly Agree”). The frequency of using team science strategies for Interpersonal Relations Skills was measured on a separate five-point scale: 1 (“Never”), 2 (“Rarely”), 3 (“Sometimes”), 4 (“Often”), and 5 (“Always”). Team Planning and Managing a Team were assessed using a familiarity scale because these areas introduced new concepts to participants, necessitating an evaluation of their understanding. In contrast, Interpersonal Relations competencies were measured using a frequency-of-use scale, as participants were presumed to be already acquainted with these concepts; the focus was thus on how often they applied these skills to enhance collaboration.

### Data analysis

We applied principal component analysis (PCA) to confirm underlying factors in participants’ responses across three Team Science skill domains. PCA is a statistical technique that reduces the dimensionality of complex datasets by transforming correlated variables into a smaller set of uncorrelated variables, called principal components, which retain most of the original information. Using factor scores based on the three components, the study examined whether self-reported teaming skills improved significantly, as determined by paired t-tests. Pre-program scores were subtracted from post-program scores to determine the impact of the CCTSI team science training program. While the dataset was nearly complete, pairwise deletion was applied to the few missing data points. All surveys were analyzed using STATA 18.

## Results

### Participant characteristics

The response rate for the pre-program survey was 53% (117 of 221 registered participants). All participants completed the online prerequisite Teaming 101 web module, and 46 out of 81 participants who completed all six workshops responded to the post-program survey, yielding a response rate of 57%. Only individuals who completed all six workshops are included in this analysis. The post-program survey had fewer respondents (*n* = 46) than the pre-program survey (*n* = 117), resulting in a notable drop-off in participation (see Table 2 for comparison).

**Table 2.** Demographics of team science training participants in the sample for pre-and post-program assessments

	Pre workshop		Post workshop	
	**N**	**%**	**N**	**%**
**Total participation**	117		46	
Cohort 1 2020	63	53.85	31	67.39
Cohort 2 2021	11	9.40	8	17.39
Cohort 3 2022	43	36.75	7	15.22
**Participant role**				
Graduate student	35	29.91	14	30.44
Postdoctoral trainee	30	25.64	10	21.74
Staff	16	13.68	8	17.39
Faculty	36	30.77	14	30.43
**Highest degree completed**				
Bachelor	11	9.40	4	8.70
Master	30	25.64	30	26.09
Doctorate	76	64.96	12	65.22
**Gender**				
Female	86	73.50	37	80.43
Male	31	26.50	9	19.57
**Race**				
White	92	78.63	36	78.26
Native American or Alaskan	1	0.85	1	2.17
Native Hawaiian or Pacific Islander	1	0.85	1	2.17
Asian	15	12.82	7	15.22
Black	3	2.56	0	0
Mixed	2	1.71	1	2.17
**Ethnicity**				
Hispanic	8	6.84	0	0
Non-Hispanic	108	92.31	46	100
**Disability**				
Disability	6	5.13	1	2.17
**First generation**				
First generation	28	23.93	5	10.87
**Low income**				
Low income	11	9.40	4	8.70

The sample comprised three cohorts from 2020 to 2022. The Team Science training program participants included graduate students, postdocs, instructors, assistant professors, clinical research professionals, research coordinators, and research assistants. The career stages of the respondents varied, with the categories being faculty (31%), graduate students (30%), postdoctoral trainees (26%), and clinical research professional staff (14%). Most participants held a doctoral degree (65%) and were female (74%). Most participants were White (78%) and of non-Hispanic descent (92%), which is comparable to the overall demographics of Colorado [21]. Five percent of participants reported living with a disability, 24% were first-generation students, and 9% were from a low-income family.

### Performance characteristics of the tool

To assess how well the TEAMS assessment tool fulfills its function – measuring Team Science competencies and organizing related skills into meaningful categories – we conducted PCA. This statistical method helps identify patterns by grouping related skills into clusters. Using orthogonal varimax rotation; we found that all items load on the respective components of Team Planning, Managing a Team, or Interpersonal Relationships with eigenvalues of 2.97, 2.77, and 2.12, respectively, well over the commonly used cut-off score of 1 [22]. Each component demonstrated strong internal consistency, indicating that the skills within each category are highly related and measure the same underlying competency. These three components explain 23%, 21%, and 16% of the variance. All the items loaded above the commonly used cut-off score of 0.3 on their respective component, and none loaded on more than one component, indicating a clear and distinct factor structure, where each item is strongly associated with a single underlying competency domain. Such a structure enhances the interpretability and validity of the assessment tool, confirming that it effectively measures the intended Team Science competencies. Higher loadings suggest that an item aligns more closely with that specific domain, meaning it contributes significantly to measuring that competency. Furthermore, Cronbach’s’ alpha – which assesses how reliably the set of items within each category measures the same underlying construct – suggests excellent internal consistency for all three components with scale reliability coefficients of 0.84 for the Team Planning component, 0.74 for the Team Managing component, and 0.81 for the Interpersonal Relations component (see Table 3). These scores are above the commonly recommended minimum value of 0.7 for scale reliability [23].

**Table 3.** Team planning, managing a team, and interpersonal relations components with corresponding loadings (orthogonal varimax rotation) and cronbach’s alpha (*α*) scale reliability coefficient

	Team Planning (familiarity)	Managing a Team (familiarity)	Interpersonal Relations (frequency)
***Team Planning α =0.84***			
Shared language	**0.42**	0.01	0.07
Vision	**0.42**	0.20	−0.12
Ground rules	**0.31**	0.11	0.03
Team charters	**0.51**	−0.08	−0.03
Navigating change	**0.49**	−0.08	0.09
***Managing a Team α =0.74***			
Meeting agendas	−0.13	**0.49**	0.15
Monitoring progress	0.01	**0.48**	0.07
Authorship agreements	0.11	**0.54**	–0.29
Incorporating other’s perspectives	−0.03	**0.41**	0.10
***Interpersonal Relations α =0.81***			
Perspective taking	−0.08	0.04	**0.46**
Awareness	0.05	−0.05	**0.51**
Diversity	0.02	−0.05	**0.49**
Turn-taking	0.00	0.17	**0.33**
**Eigenvalue**	2.97	2.77	2.12
**Variance explained**	0.23	0.21	0.16

*Note:* Values greater than **0.30** are bold and indicate an association with one of the three components.

**Figure 1.**
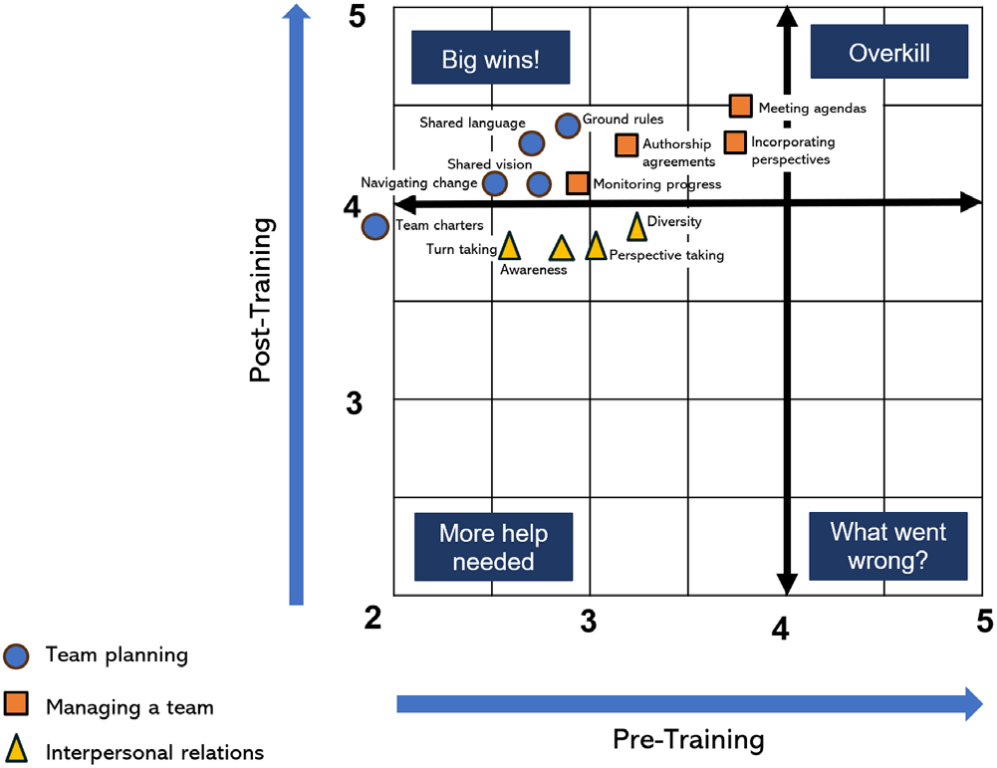
Mean pre- and post-program 5-item likert scores for variables related to team planning (Familiarity), managing a team (Familiarity), and interpersonal relationship (Frequency) skills.

Mean pre- and post-program familiarity scores and frequency of Team Science strategy use values were plotted on a four-quadrant graph to demonstrate the effectiveness of the training on perceived familiarity and frequency of use regarding the core competencies. These visualizations are organized with the pre-scores on the *X*-axis and the post-scores on the *Y*-axis. Low pre- and post-program scores would indicate that more Team Science training is needed, while low pre- and high post-program scores suggest that the workshops substantially improved team science capability. High pre- and post-program scores suggest that information and skill development may have been overkill, as participants were already proficient, as indicated by their self-reports. Finally, if the pre-program scores were high, but the post-program scores were low, it could suggest that the training program was ineffective, or participants initially overestimated their ability before the program (see Figure 1).

### Impact of team science training by competency domains

The four-quadrant graph (see Figure 1) shows that the training achieved “big wins” for the Team Planning and Managing Team competency domains. Familiarity with Team Planning skills showed the greatest gains from pre- to post-program, with the mean shifting from 1 to 2 total points (see Table 4). Four items in the Team Planning component (shared language, shared vision, creating ground rules, and navigating change) scored below 3 in the pre-program survey and over 4 in the post-program survey (see Table 4 for mean scores). The fifth item, creating team charters, showed the greatest change, where participants initially indicated little familiarity (1.9) and reported substantially stronger familiarity (3.9) after the workshop series.

**Table 4.** Mean pre- and post-program scores with standard deviations

	Mean pre-program	Mean post-program
***Team Planning* (familiarity)**		
Shared language	2.61 (1.11)	4.23 (.63)
Shared vision	2.74 (1.10)	4.06 (.67)
Ground rules	2.96 (1.08)	4.40 (.64)
Creating team charters	1.88 (1.03)	3.91 (.80)
Navigating change	2.51 (1.05)	4.08 (.65)
***Managing a Team* (familiarity)**		
Creating meeting agendas	3.72 (.99)	4.51 (.59)
Monitoring progress	2.91 (1.04)	4.06 (.63)
Establishing authorship agreements	3.21 (1.19)	4.34 (.81)
Incorporating other’s perspectives	3.79 (.97)	4.31 (.66)
***Interpersonal Relations* (frequency)**		
Perspective taking	3.05 (3.05)	3.78 (.90)
Awareness	2.87 (1.10)	3.76 (.83)
Diversity	3.23 (1.05)	3.87 (.71)
Turn-taking	2.66 (1.21)	3.76 (1.14)

*Note*: all values are rated on a five-point Likert scale ranging from strongly disagree (1) to strongly agree (5) to measure familiarity (Team Planning, Managing a Team) or frequency of using strategies when working on a research project (1) never to (5) always (and Interpersonal Relations) pre- and post-program.

All four items in the Managing a Team competency (creating meeting agendas, monitoring progress, establishing authorship agreements, incorporating others’ perspectives) were also in the “big wins” quadrant. The items associated with this component had higher pre-program scores than the Team Planning component. Overall, these items showed moderate pre- and high post-program scores as a cluster and suggested that skills associated with Managing a Team can be effectively taught in a short virtual workshop setting. These results show “big wins” as a cluster for both the Team Planning and Managing a Team components, suggesting that the team science training effectively increased collaborative capacity across these two core competency domains.

The items in the Interpersonal Relations component showed more moderate increases, ranging from 0.64 to 1.1, compared to the more extensive growth or final means above 4.0 in the Team Planning and Managing a Team components. Participants’ pre-program results in this competency range from 2.66 to 3.23, indicating an opportunity for skill development through targeted training. While scores on all items increased in the post-program assessment (*p* values <0.05), the average score for all items remained slightly below the optimal outcome (see Table 4).

## Conclusions

Results indicate that the CCTSI Team Science training program significantly (*p* =< 0.05) increased the self-reported teaming competency of participants. The three distinct, empirically confirmed components – Team Planning, Managing a Team, and Interpersonal Relations – from the TEAMS survey instrument presents a novel contribution to team science training, evaluation, and team science literature. While prior research has emphasized the importance of communication, coordination, and trust within teams, these specific groupings and their operationalization through validated survey items have not been formalized. Using PCA with strong reliability coefficients (α = 0.84, 0.74, and 0.81, respectively), this study provides initial evidence of construct validity for evaluating team science competencies. Furthermore, pre- and post-program results (Table 4) show substantial gains across all three domains, particularly in Team Planning, where familiarity with practices such as creating team charters and establishing ground rules nearly doubled. These findings suggest an opportunity for other CTSA hubs and interdisciplinary research training programs to adopt this structure as a framework for evaluating team science initiatives. By incorporating clear domains and validated metrics, programs can more systematically assess where participants are starting from, how their familiarity with and use of practices evolve, and where additional support is needed. The approach also fills a gap in the current evaluation landscape by offering a flexible, evidence-based tool for measuring familiarity and frequency of use for essential team science strategies.

In the assessment of the Team Science Training program, we found that the Team Planning and Managing a Team competencies may have achieved the most substantial self-reported gains because they involve structured, explicit knowledge and tangible activities, such as authorship agreements and meeting agendas, that are easier to implement in practice. Participants also reported statistically significant improvements in the development of Interpersonal Relationship competency. However, the mean scores did not reach the desired application levels, as measured by the frequency of “most of the time” application on research projects. This finding suggests that more and/or different training may be needed to enhance Interpersonal Relationship skills, such as increased role-playing activities or exercises to practice these skills in real-life situations. Considering these findings, we recommend that future training programs for teaming assess explicit and tacit collaboration skills, which are often overlooked in traditional evaluations. These programs should consider developing context- and team-specific interventions and training methods tailored to the unique dynamics of each team [10]. By doing so, organizations can better equip teams with the visible and underlying skills necessary for effective collaboration.

This study highlights that participant significantly increased their self-assessed Interpersonal Relationship skills, there is still more room for growth in these competencies. Further development may require greater emphasis and integration of learning tools that deepen interpersonal skills and promote self-reflection. Future training efforts may benefit from involving the entire team in practicing interpersonal skills in a more context-specific setting or incorporating more relationship-building activities, whether virtual or in-person [24]. Team Science training should actively engage with cross-cultural differences and multi-layered identities. It could benefit from considering established practices in intercultural communication training to enhance collaborative capacity and foster an inclusive environment [25,26]. To increase innovation in research teams by broadening participation, Team Science program instructors must acknowledge and create a safe space in workshops to discuss personal experiences, identify targeted strategies to foster communication across interdisciplinary differences, and explore cultural differences [25,27]. Sharing experiences can be validating for those who have faced similar encounters and raise awareness for those who haven’t. Furthermore, Team Science program instructors should collect and share the experiences and strategies of individuals with similar demographics who have overcome uncollaborative settings to empower self-efficacy in participants. In addition, scientists on teams should familiarize themselves with both on-campus resources, such as academic advisors, university counseling services, and research offices, as well as off-campus resources, including professional organizations, mental health professionals, or specialized consultants, in case a teaming challenge arises that could benefit from professional assistance. Finally, the faculty of Team Science training programs should consider tailoring the program with examples relevant to specific demographics to acknowledge persistent inequalities, create a safer space, foster social support, and promote solidarity.

### Limitations and directions for future research

This study has several important limitations that should be considered when interpreting and applying the findings. First, the post-program survey responses were lower (*n* = 47) than the pre-program survey response rate (*n* = 117), which could introduce response bias. The high attrition rate underscores the need for future studies to explore strategies for improving participant retention. Additionally, using self-reported measures, while helpful in capturing participant perceptions, may be influenced by cognitive biases, where individuals either overestimate or underestimate their familiarity and frequency of use. Despite these limitations, the results suggest that participants found value in Team Science training. Self-reported improvements were observed in key teaming aspects, such as Team Planning, Managing a Team, and Interpersonal Relationship skills. The increase in participants’ perceived teaming competencies suggests that these general skills can be reinforced through active learning workshops focused on skills identified in the literature [5,12].

Further research is needed to explore how phenomena like the Matthew Effect – where individuals or groups with existing advantages, such as prior leadership experience or access to resources, tend to gain even more recognition and opportunities – might influence self-reported outcomes in team science training programs. This is particularly important for participants from historically underrepresented groups, who may face unique structural challenges, such as limited access to resources, which can affect both their experiences in the program and their perception of progress. Future studies might consider how cognitive biases, such as the Dunning-Kruger Effect, where individuals may overestimate or underestimate their skills, might impact participants’ self-assessments and inform the development of new measures for Team Science competencies that don’t rely exclusively on self-assessment. To ensure that future findings are more robust and generalizable, additional research should prioritize recruitment and retention strategies that enhance the diversity and representation of participants throughout the study. Larger and more diverse cohorts of early-career researchers and clinical research professionals, combined with behavioral observations, are crucial for validating these findings and further investigating how these biases and structural inequities impact outcomes.

## References

[1] Lin CY , Huang CK. Employee turnover intentions and job performance from a planned change: the effects of an organizational learning culture and job satisfaction. Int J Manpow. 2021;42(3):409–423. doi: 10.1108/IJM-08-2019-0390.

[2] Park KA , Johnson KR. Job satisfaction, work engagement, and turnover intention of CTE health science teachers. Int J Res Vocat Educ Train. 2019;6(3):224–242. doi: 10.13152/IJRVET.6.3.4.

[3] Mendell A , Fritter J , Helm S , et al. Team science competencies for clinical research professionals: a multi-leveled Delphi approach. J Clin Transl Sci. 2024;8:1–25. doi: 10.1017/cts.2024.509. PMC11626577 39655002

[4] Mayowski CA , Norman MK , Schenker Y , Proulx CN , Kapoor WN. Developing a team science workshop for early-career investigators. J Clin Transl Sci. 2019;3(4):184–189. doi: 10.1017/cts.2019.394. 31660242 PMC6799325

[5] Bisbey TM , Wooten KC , Campo MS , Lant TK , Salas E. Implementing an evidence-based competency model for science team training and evaluation: TeamMAPPS. J Clin Transl Sci. 2021;5(1):e142. doi: 10.1017/cts.2020.598. 34422322 PMC8358845

[6] Fiore SM , Gabelica C , Wiltshire TJ , Stokols D. Training to be a (team) scientist. In: Hall KL , Vogel AL , Croyle RT , ed. Strategies for Team Science Success. Springer International Publishing, 2019: 421–444. doi: 10.1007/978-3-030-20992-6_33.

[7] Hall KL , Vogel AL , Croyle RT , eds. Strategies for Team Science Success: Handbook of Evidence-Based Principles for Cross-Disciplinary Science and Practical Lessons Learned from Health Researchers. Springer International Publishing, 2019. doi: 10.1007/978-3-030-20992-6.

[8] Hall KL , Vogel AL , Huang GC , et al. The science of team science: a review of the empirical evidence and research gaps on collaboration in science. Am Psychol. 2018;73(4):532–548. doi: 10.1037/amp0000319. 29792466

[9] Love HB , Fosdick BK , Cross JE , et al. Towards understanding the characteristics of successful and unsuccessful collaborations: a case-based team science study. Humanit Soc Sci Commun. 2022;9(1):371. doi: 10.1057/s41599-022-01388-x.

[10] Linhardt RM , Bisbey TM , Salas E. The science and practice of team training: historical progress and a research agenda. Consult Psychol J. 2024;76(1):70–92. doi: 10.1037/cpb0000263.

[11] Salas E , Reyes DL , McDaniel SH. The science of teamwork: progress, reflections, and the road ahead. Am Psychol. 2018;73(4):593–600. doi: 10.1037/amp0000334. 29792470

[12] Lotrecchiano GR , DiazGranados D , Sprecher J , et al. Individual and team competencies in translational teams. J Clin Transl Sci. 2021;5(1):1–5. doi: 10.1017/cts.2020.551. PMC8057415 33948290

[13] Rolland B , Cross JE , Hohl SD , Johnson LJ , Wooten K , Brasier AR. Introduction to the themed issue on the design, development, evaluation, and dissemination of team science interventions in clinical and translational research. J Clin Transl Sci. 2021;5(1):1–5. doi: 10.1017/cts.2020.584. PMC8727717 35047214

[14] Read EK , O’Rourke M , Hong GS , et al. Building the team for team science. Ecosphere. 2016;7(3):1–9. doi: 10.1002/ecs2.1291.

[15] Abu-Rish Blakeney E , Kang S , Henrikson K , et al. Implementation and evaluation of team science training for interdisciplinary teams in an engineering design program. J Clin Transl Sci. 2021;5(1):1–10. doi: 10.1017/cts.2021.788. PMC8327544 34367672

[16] Duff D , Tomblin JB , Catts H. The influence of reading on vocabulary growth: a case for a Matthew effect. J Speech Lang Hear Res. 2015;58(3):853–864. doi: 10.1044/2015_JSLHR-L-13-0310. 25812175 PMC4610292

[17] Petersen AM , Jung WS , Yang JS , Stanley HE. Quantitative and empirical demonstration of the Matthew effect in a study of career longevity. Proc Natl Acad Sci. 2011;108(1):18–23. doi: 10.1073/pnas.1016733108. 21173276 PMC3017158

[18] Bradley CS , Dreifuerst KT , Johnson BK , Loomis A. More than a meme: the Dunning-Kruger effect as an opportunity for positive change in nursing education. Clin Simul Nurs. 2022;66:58–65. doi: 10.1016/j.ecns.2022.02.010.

[19] Fitzmaurice S. Educational interpreters and the Dunning-Kruger effect. J Interpret. 2020;28(2):1.

[20] Arikan S , Dochy F , Segers M. Framing the effects of high-impact practices from a high-impact learning perspective. A review of studies. Creat Educ. 2022;13(9):2994–3025. doi: 10.4236/ce.2022.139190.

[21] Colorado Demography Office. Demography. Colorado Department of Local Affairs website. (https://demography.dola.colorado.gov/) Accessed November 2, 2024.

[22] Kline TJ. Psychological testing: A practical approach to design and evaluation. Sage Publications, 2005. (https://books.google.com/books?hl=en&lr=&id=FtNyAwAAQBAJ&oi=fnd&pg=PA1&dq=kline+psychological+testing&ots=n_TXNXtgRW&sig=KRXnSmpneLSPS4Nx88jaJzH2-8A) Accessed November 23, 2023.

[23] Bujang MA , Omar ED , Baharum NA , et al. A review on sample size determination for Cronbach’s alpha test: A simple guide for researchers. Malays J Med Sci. 2018;25(6):85–99. doi: 10.21315/mjms2018.25.6.9. 30914882 PMC6422571

[24] Lacerenza CN , Marlow SL , Tannenbaum SI , Salas E. Team development interventions: evidence-based approaches for improving teamwork. Am Psychol. 2018;73(4):517–531. doi: 10.1037/amp0000295. 29792465

[25] Feitosa J , Hagenbuch S , Patel B , Davis A. Performing in diverse settings: a diversity, equity, and inclusion approach to culture. Int J Cross Cult Manag. 2022;22(3):433–457. doi: 10.1177/14705958221136707.

[26] Perry KJ , Mutignani LM , Gette JA , et al. The paper chase: A team science training exercise. Train Educ Prof Psychol. 2024;18:13–20. doi: 10.1037/tep0000448. 38487794 PMC10936699

[27] O’Rourke M , Rinkus MA , Cardenas E , McLeskey C. Communication practice for team science. In: Gosselin D , ed. A Practical Guide for Developing Cross-Disciplinary Collaboration Skills. Springer International Publishing, 2023: 83–102. doi: 10.1007/978-3-031-37220-9_5.

[28] Putman VL , Paulus PB. Brainstorming, brainstorming rules and decision making. J Creat Behav. 2009;43(1):29–40. doi: 10.1002/j.2162-6057.2009.tb01304.x.

[29] Edmondson A. Psychological safety and learning behavior in work teams. Adm Sci Q. 1999;44(2):350–383. doi: 10.2307/2666999.

[30] Dietsch AM , Wald DM , Stern MJ , Tully B. An understanding of trust, identity, and power can enhance equitable and resilient conservation partnerships and processes. Conserv Sci Pract. 2021;3(6):e421. doi: 10.1111/csp2.421.

[31] Bender L , Walia G , Kambhampaty K , Nygard KE , Nygard TE. Social sensitivity correlations with the effectiveness of team process performance: An empirical study. In: Proceedings of the Ninth Annual International Conference on International Computing Education Research. 2012: 39–46. doi: 10.1145/2361276.2361285.

[32] Brown B. Atlas of the Heart: Mapping Meaningful Connection and the Language of Human Experience. Random House, 2021.

[33] Woolley AW , Chabris CF , Pentland A , Hashmi N , Malone TW. Evidence for a collective intelligence factor in the performance of human groups. Science. 2010;330(6004):686–688. doi: 10.1126/science.1193147. 20929725

[34] Rasmussen LM , Banks G , Demeter E , et al. Authorship agreements benefit researchers and research culture. Nat Hum Behav. 2023;7(12):2044–2045. doi: 10.1038/s41562-023-01758-8. 37945808

[35] Mohammed S , Harrison DA. The clocks that time us are not the same: a theory of temporal diversity, task characteristics, and performance in teams. Organ Behav Hum Decis Process. 2013;122(2):244–256. doi: 10.1016/j.obhdp.2013.08.004.

[36] Nissani M. Fruits, salads, and smoothies: a working definition of interdisciplinarity. J Educ Thought. 1995;29(2):121–128. doi: 10.55016/ojs/jet.v29i2.52385.

[37] Eigenbrode SD , O’Rourke M , Wulfhorst JD , et al. Employing philosophical dialogue in collaborative science. BioScience. 2007;57(1):55–64. doi: 10.1641/B570109.

[38] Salazar MR , Widmer K , Doiron K , Lant TK. Leader integrative capabilities: A catalyst for effective interdisciplinary teams. In: Hall KL , Vogel AL , Croyle RT , eds. Strategies for Team Science Success: Handbook of Evidence-Based Principles for Cross-Disciplinary Science and Practical Lessons Learned from Health Researchers. Springer, 2019: 313–328. doi: 10.1007/978-3-030-20992-6_24.

[39] McFadzean E. Techniques to enhance creative thinking. Team Perform Manag. 2000;6:62–72. doi: 10.1108/13527590010731989.

[40] Hubbs G , O’Rourke M , Orzack SH , eds. The Toolbox Dialogue Initiative: The Power of Cross-Disciplinary Practice. CRC Press, 2020.

[41] Rinkus MA , Donovan SM , Hall TE , O’Rourke M. Using a survey to initiate and sustain productive group dialogue in focus groups. Int J Soc Res Methodol. 2021;24(3):327–340. doi: 10.1080/13645579.2020.1786240.

[42] McKeown G. Essentialism: The Disciplined Pursuit of Less. Crown Currency, 2014. doi: 10.48558/xtb1-2820.

[43] Thomas K , Kilmann R. An Overview of the Thomas-Kilmann Conflict Mode Instrument. Kilmann Diagnostics, 2007. www.kilmanndiagnostics.com/overview-thomas-kilmann-conflict-mode-instrument-tki (Accessed June 14, 2025).

[44] Souza T. Managing hot moments in the classroom: Concrete strategies for cooling down tension. Diversity and Inclusion in the College Classroom, 2018. https://www.facultyfocus.com/articles/effective-classroom-management/responding-to-microaggressions-in-the-classroom/ (Accessed June 14, 2025).

[45] Thompson G , Vecchio RP. Situational leadership theory: a test of three versions. Leadersh Q. 2009;20(5):837–848. doi: 10.1016/j.leaqua.2009.06.014.

[46] Ford J , Harding N. Followers in leadership theory: Fiction, fantasy and illusion. Leadership. 2018;14(1):3–24. doi: 10.1177/1742715015621372.

[47] Mullen CA , Klimaitis CC. Defining mentoring: a literature review of issues, types, and applications. Ann N Y Acad Sci. 2021;1483(1):19–35. doi: 10.1111/nyas.14176. 31309580

